# Systematic Review on the Safety of Outpatient Upper Airway Surgery for Obstructive Sleep Apnea Patients in Ambulatory Surgical Centers

**DOI:** 10.1177/19160216251379325

**Published:** 2025-10-29

**Authors:** Muaaz Asghar, Kenny Pang, Mauz Asghar, Brian Rotenberg

**Affiliations:** 1 College of Medicine, University of Saskatchewan, Saskatoon, SK, Canada; 2 Asia Sleep Centre, Singapore, Singapore; 3 Department of Otolaryngology—Head and Neck Surgery, Schulich School of Medicine and Dentistry, London, ON, Canada

**Keywords:** obstructive sleep apnea, OSA, sleep apnea, ambulatory surgical centers, outpatient, outpatient surgery, day-case surgery, ambulatory surgery

## Abstract

**Importance:**

This systematic review determines that the patients with obstructive sleep apnea (OSA) can undergo upper airway surgery in ambulatory surgical centers (ASCs) safely and determines which patients with OSA are appropriate for this environment.

**Objective:**

The systematic review aimed to determine the safety of conducting upper airway surgeries on patients with OSA in ASCs.

**Design:**

The Preferred Reporting Items for Systematic Reviews and Meta-Analyses guidelines were followed to perform a systematic review on ambulatory upper airway surgery studies. A comprehensive search was conducted from MEDLINE, Embase, CENTRAL, and Scopus from inception through February 2024. A descriptive analysis was conducted. Risk of bias was assessed using the Murad Tool and the Newcastle-Ottawa Scale.

**Setting:**

Hospital outpatient department or ambulatory surgical center.

**Participants:**

Adult patients with OSA.

**Intervention:**

Upper airway surgery.

**Main Outcome Measures:**

Unplanned admission rates and 24 hour complications.

**Results:**

From 9313 studies, 11 upper airway surgery studies with 5714 participants were identified. Studies observed a 5.4% admission rate for medical reasons, predominantly stemming from avoidable admissions for desaturations. There was a 9.2% 24 hour complication rate. By initiating an oxygen discontinuation trial, OSA patients with controlled comorbidities can confidently proceed with OSA surgery at ASCs feasibly and safely. Patient selection is paramount in the ASC environment, with a focus on age, body mass index, apnea-hypopnea index, and controlled comorbidities.

**Conclusions and Relevance:**

OSA patients with mild or controlled comorbidities can safely undergo ambulatory OSA surgery in ASCs without sacrificing the cost-effectiveness of the ASC model. Future studies should use larger populations and prospective study designs.

**Other:**

The protocol for this review was registered with the PROSPERO database (registration number: CRD42023415162).

## Graphical abstract

**Graphical Abstract:**
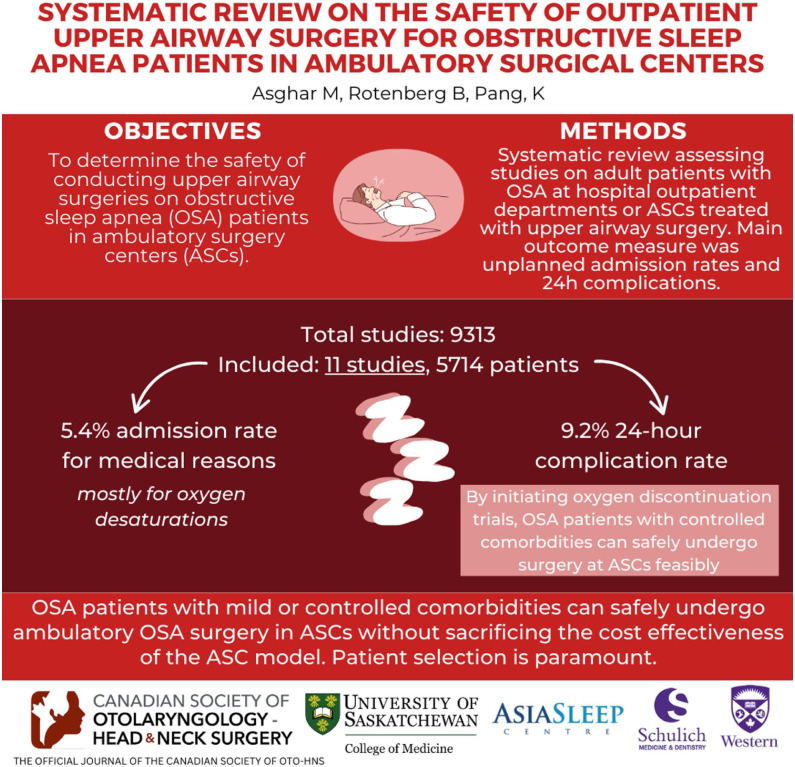

## Key Messages

1. Nasal/palatopharyngeal surgery can be performed in an outpatient center in the ambulatory surgical center setting with appropriate patient selection.
2. Patient selection should be made on age, body mass index, apnea-hypopnea index, and controlled comorbidities.

## Introduction

Obstructive sleep apnea (OSA) is the most common sleep disorder, afflicting up to 38% of adults.^1^ Airway resistance due to soft tissue pliability and extrinsic factors, such as body mass index (BMI), can prompt airway collapse which can lead to apneas and subsequent awakenings.^2^ These disturbances can cause systemic issues such as hypertension, stroke, insulin resistance, and a risk for early-postoperative cardiopulmonary complications.^3-5^ Consequently, the suitability of patients with OSA for outpatient surgery was historically debated.^6^

A consensus statement from the Society for Ambulatory Anesthesia from 2012 has indicated that patients with well-controlled OSA and comorbidities can undergo outpatient surgery in a hospital setting. However, when this statement was issued, there were limited data on subjects and no specific conclusions were made regarding nasal/palatopharyngeal (NPP) surgery due to lack of data.^7^ However, recent research has indicated that NPP surgery can be performed safely in an outpatient environment. This is evident as the most common complication of upper airway surgery for OSA has been found to be respiratory and large majority of these respiratory complications have been simple desaturations with events necessitating intervention being minimal, implying limited need for overnight monitoring.^8^ Additionally, Tan et al’s systematic review provides further evidence supporting the feasibility of routine outpatient NPP surgery for selected patients with OSA.^9^

In 2009, over 53 million outpatient surgeries were performed in the United States, indicating their increasing prevalence.^10^ To accommodate for rising demand, cost-saving ambulatory surgical centers (ASCs) were established, enabling surgeries outside of hospital settings, thus reducing the burden on a strained health care system.^11^ The patient selection criteria for care at ASCs are different from a hospital outpatient department because ASCs have limited resources to handle severe complications, and their cost-saving model becomes moot if patients were routinely transferred for overnight admissions. Therefore, proper selection of patient and surgery is necessary for in an ASC.^12^

While some patients with OSA have been found to be safe to undergo outpatient surgery in a hospital, no systematic reviews have assessed their risk in an ASC environment for NPP surgery. Additionally, there is an unanswered question regarding the feasibility of primary OSA surgery at an ASC setting. The review aimed to address this deficiency in the literature. To accomplish this, we addressed the following question: Is the ASC environment safe for NPP surgeries for patients with OSA?

## Materials and Methods

A systematic review was conducted in accordance with the Preferred Reporting Items for Systematic Reviews and Meta-Analyses (PRISMA) statement.^13^ A comprehensive search of Ovid-MEDLINE, Ovid-Embase, Cochrane, and Scopus was conducted from inception to February 2024 with the assistance of a librarian (Supplemental Tables 1–4).

Title and abstracts were deduplicated by Rayyan (https://www.rayyan.ai/) and were then independently examined by 2 authors (M.A., M.U.A.) against predefined criteria (Table 1). The studies must encompass upper airway surgeries specifically for patients with OSA. We define upper airway surgery as any surgery involving the nose (septoplasty, rhinoplasty, turbinate reduction, sinus surgery) or palate/pharynx (variants of palatoplasty, tonsillectomy, tongue-base surgery). Multilevel surgery is defined as a surgery including multiple palatopharyngeal operations or a combination of a nasal operation with a palatopharyngeal operation. Notably, hypoglossal nerve stimulation does not fit into this category, as it directly treats sleep apnea without altering the upper airway. Given its distinct surgical approach, we have opted to exclude it to concentrate on the feasibility of NPP surgery.

**Table 1.** Inclusion and Exclusion Criteria.

Inclusion criteria	Population	>10 OSA patients identified with a questionnaire, PSG, self-report, or preoperative records who are 18 y or older that are going outpatient OSA surgery
	Intervention	Outpatient upper airway surgery used to treat OSA, excluding hypoglossal nerve stimulation
	Comparison	Not necessary but can be patients with OSA who are 18 y or older that have undergone inpatient surgery
	Outcome	Primary outcome is unplanned admissions and 24 h complications Secondary outcome is 30 d complications/readmission/ER visits and mortality rate
	Study design	Retrospective or prospective studies Case series, cohort, case-control, and RCTs
Exclusion criteria		Conference abstracts, pediatric population, reviews, questionnaires, local anesthetic with no sedation used, expert opinions, recommendations, animal subjects, non-English

Abbreviations: ER, emergency room; OSA, obstructive sleep apnea; PSG, polysomnography; RCT, randomized control trial.

Once relevant abstracts were identified, full text-articles were assessed. Any discrepancies were discussed and resolved via consensus. Initial data collection was conducted by one author (M.U.A.) and was independently confirmed by another (M.A.). The primary outcomes were 24 hour complications and unplanned admissions. The following characteristics were also collected for each study: first author, year, study design, number of outpatients/inpatients, number of OSA/non-OSA patients, participant characteristics, selection and admission protocol, type of surgery performed, 30 day postoperative complications, unplanned admission causes, follow-up length, factors for unplanned admissions or postoperative complications, and surgical setting.

The review lacked ASC studies, making the outpatient group a less effective benchmark for this review’s objectives. Outpatient surgeries based in hospitals might have higher admissions due to reasons like societal factors, organizational factors, and anesthetic complications. However, such reasons would have little to no bearing in the ASC setting. Additionally, admissions for respiratory monitoring were not accounted for, as patients with no complications in the post-anesthesia care unit (PACU) typically do not experience severe complications overnight.^8,14^ Moreover, ASCs prefer younger and healthier patients, making it less likely for them to require respiratory monitoring unless there’s a specific medical concern. Relying solely on raw unplanned admissions as a metric for ASC practice might skew the interpretation. As such, within the OSA surgery review, only patients admitted due to medical complications were factored in. Other studies with invasive surgeries within ASC report unplanned admission rate from 0.0% to 2.3%.^15-17^ Since outpatient surgeries in hospitals will have candidates that are likely older and less healthy than the candidates for ASCs, we put a benchmark of 3.0% admission rate for medical causes within this review as being a key threshold.

The risk-of-bias assessment was performed by a single author (M.A.). The Cochrane Risk of Bias Tool, the Newcastle-Ottawa Scale (NOS), and Murad Tool were used to look at randomized controlled trials, cohort/case-control studies, and case series studies, respectively.^18-20^ For NOS, 8 to 9 score was considered high quality, 6 to 7 was considered moderate quality, and 5 or less was considered low quality. The protocol for this review was registered with the PROSPERO database (registration number: CRD42023415162).

Pooled summary statistics were calculated for patient characteristics, unplanned hospital admission rates, 24 hour and 30 day postoperative complication rates. Median was used for the calculation of the average when a mean was not present.

## Results

**Figure 1.**
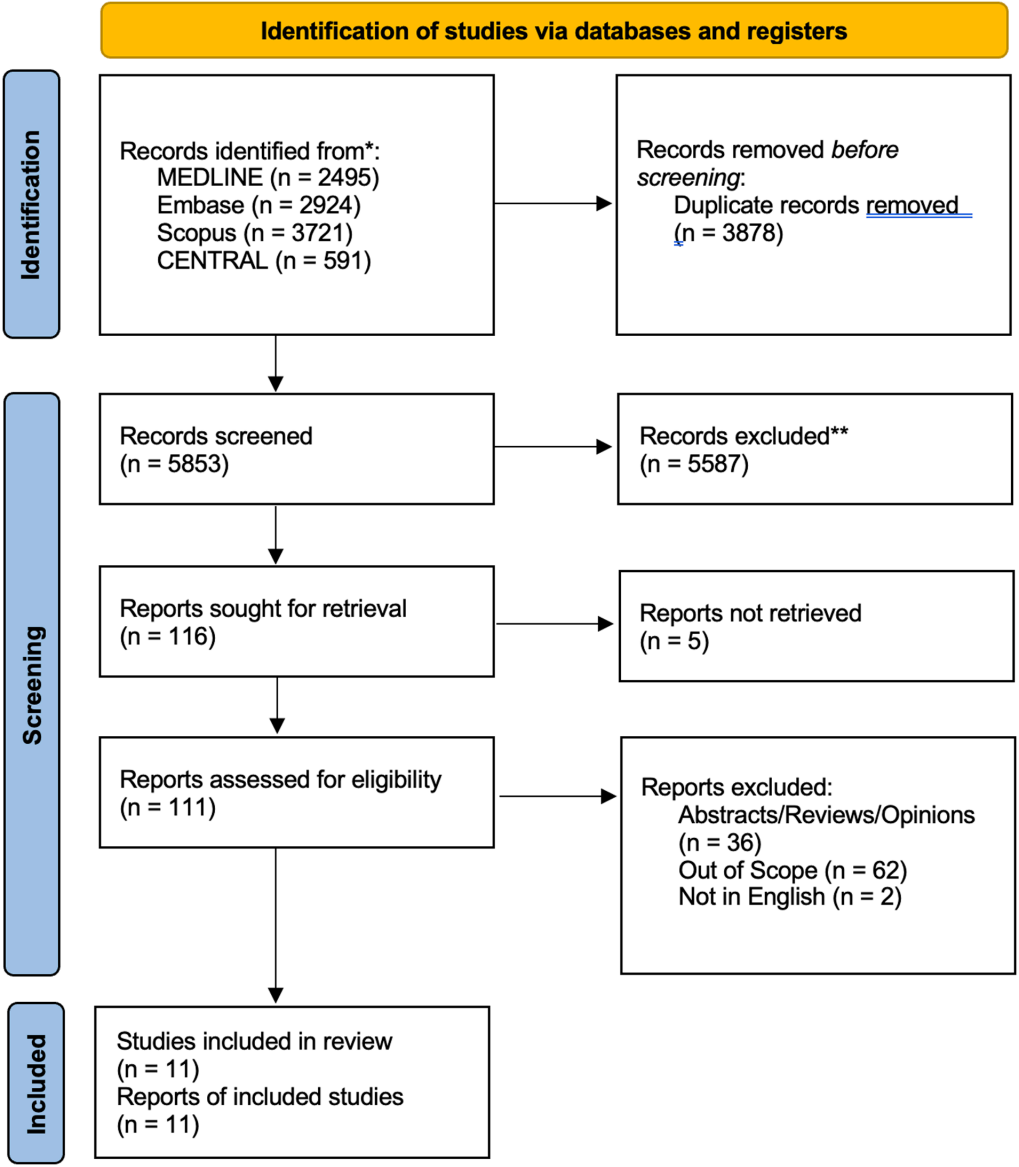
PRISMA flow diagram. PRISMA, Preferred Reporting Items for Systematic Reviews and Meta-Analyses.

Our searches yielded 9731 articles, from which 3878 were found to be duplicates and removed. Following a thorough review of titles and abstracts, 111 full-text articles were examined. Ultimately, 11 studies met the inclusion criteria.^21-31^ Figure 1 uses the PRISMA flow diagram to illustrate the search and selection process. Tables 2 to 5 present the risk of bias, characteristics, and outcomes of the included studies.

**Table 2.** Risk of Bias Tool for Case Series Using Murad Tool.

Studies	Selection	Ascertainment		Causality	Reporting
Bhattacharyya^22^	✓	✓	✓	✓	✓
Hathaway and Johnson^23^	✓	✓	0.5	✗	✓
Rotenberg et al^27^	✗	✓	✓	✓	✓
Stefanescu et al^30^	✗	✓	✗	✓	✗
Strocker et al^29^	✓	✓	✓	✓	✓
Wu et al^31^	✓	✓	✓	✗	✓

**Table 3.** Risk of Bias Tool Using the Newcastle-Ottawa Scale.

Studies	Selection	Comparability	Outcome	Total
Baugh et al^21^	3/4	1/2	3/3	7/9
Kandasamy et al^24^	4/4	0/2	3/3	7/9
Kieff and Busaba^25^	2/4	2/2	3/3	7/9
Rosero and Joshi^26^	3/4	2/2	3/3	8/9
Spiegel and Raval^28^	3/4	0/2	2/3	5/9

**Table 4.** Data for Included Studies.

Study (N = number of outpatients, n = number of inpatients)	Setting/surgery performed	Age (inpatient) BMI (inpatient) AHI/RDI (in patient)	Admission rate (%)	24 h Postoperative complication rate for outpatient (%, inpatient complication rate)	30 d Complication rate (%, inpatient complication rate)	30 d Mortality (%)
Baugh et al^21^ N = 404 n = 48	Hospital/nasal, palatopharyngeal	33 (32) N/A N/A	N/A	N/A	Revisits: 19.8, admissions: 2.0 (revisits: 16.7, admissions: 2.1)	N/A
Bhattacharyya^22^ N = 2349	ASC/UPPP	44.9 N/A N/A	N/A	N/A	Revisits: 6.6, readmissions: 1.7	0.04
Hathaway and Johnson^23^ N = 110	Hospital/UPPP	44.0 32.3 RDI: 35.0	3.6	12.7	9.1 Complication, 2.7 readmissions	N/A
Kandasamy et al^24^ N = 97 n = 248	Hospital/UPPP	33.2 (36.8) 29.1 (30.1) AHI: 23.6 (35.3)	N/A	20.3 (30.3)	20.6 (31.3)	0.0 (0.0)
Kieff and Busaba^25^ N = 23 n = 63	Hospital/multilevel	45.9 (48.0) 28.7 (32.5) RDI: 36.0 (36.5)	N/A	0.0	0.0 (4.8)	0.0 (0.0)
Rosero and Joshi^26^ N = 987 n = 987	Not mentioned/airway surgery	40.6 (41.0) 31.9 (32.2) N/A	N/A	N/A	2.2 Complications, 3.6 readmissions (2.7 complications, 3.1 readmissions)	<0.1
Rotenberg et al^27^ N = 50	Hospital/palatal, nasal, radiofrequency tongue ablation, tonsillectomy	45.4 30.4 AHI: 24.4	22.0	22.0	N/A	N/A
Spiegel and Raval^28^ N = 10 n = 107	Hospital/ UPPP	N/A N/A N/A	N/A	0.0 (4.7)	N/A	N/A
Stefanescu et al^30^ N = 122	ACS/tonsillectomy, UPPP	N/A N/A N/A	N/A	0.9	0.0 Infectious and bleeding complications	N/A
Strocker et al^29^ N = 40	Hospital/UPPP	40 27.1 AHI: 39.5	2.5	2	2.5 Readmission related to lumbar disc, 0.0 revisits	0
Wu et al^31^ N = 79	Hospital/rhinological, laryngeal	51.7 N/A N/A	1.3	1.3	N/A	N/A

Abbreviations: AHI, apnea-hypopnea index; ASC, ambulatory surgical center; BMI, body mass index; N/A, not applicable; RDI, respiratory disturbance index; UPPP, uvulopalatopharyngoplasty.

**Table 5.** Relevant Findings for Included Studies.

Study	Selection criteria Discharge criteria	Cause for admission for outpatient population and inpatient population	24 h Specific complications for outpatient population and inpatient population	Relevant findings of study
Baugh et al^21^	Surgeon/anesthesiologist Surgeon/anesthesiologist	N/A	N/A	No statistically-significant difference between inpatient and outpatient, no 30 d ER visits or hospital readmissions, pain is most likely admission for outpatient and hemorrhage is next for this study, uses insurance claims instead of hospital information
Bhattacharyya^22^	Surgeon/anesthesiologist Surgeon/anesthesiologist	N/A	N/A	Hemorrhage was most likely cause of admission, pain was next most likely cause of admission, death reported but does not give time or cause
Hathaway and Johnson^23^	No severe cardiopulmonary comorbidities or apnea associated comorbidities, no skeletal surgery or tracheostomy Surgeon/anesthesiologist	75.0% Desaturations 25.0% Medication-related cause	10% Anesthetic complications (oral intake and nausea) 2.7% Desaturations	Anesthetic complications was the most likely cause of admission, finds severity of OSA does not affect admission, but does not perform a statistical analysis, uses defined selection criteria
Kandasamy et al^24^	Surgeon/anesthesiologist and hospital criteria, selection of outpatient and inpatients are based on discharge, not intention Surgeon/anesthesiologist and hospital criteria	N/A	In PACU: 4.1% desaturation, 6.0% desaturation 24 h Complications: 5.2% desaturation, 15.7% desaturation, 1.2% hemorrhage	Bias due to the selection of outpatient and inpatients are based on discharge, not intention, desaturation was considered >88% oxygen saturation, no statistically difference in PACU complications or desaturations but statistically-significant difference in 24 h complications and desaturations, likely due to overnight desaturations of inpatient. AHI and BMI were found to increase complications
Kieff and Busaba^25^	Selection of outpatient and inpatients are based on discharge, not intention, surgeon/anesthesiologist Constant normal oxygen saturation above 94%, normal oral intake and analgesia, stable vital signs, no coronary artery disease, COPD, diabetes mellitus	N/A	None	Bias due to the selection of outpatient and inpatients are based on discharge, not intention
Rosero and Joshi^26^	Selection of outpatient and inpatients are based on discharge, not intention, surgeon/anesthesiologist Surgeon/anesthesiologist	N/A	N/A	Bias due to the selection of outpatient and inpatients are based on discharge, not intention, no statistically-significant difference
Rotenberg et al^27^	No postoperative admissions for causes other than OSA, no major tongue-base surgeries (eg, lingual tonsillectomy, submucosal resection), no cardiovascular comorbidities (resistant hypertension, heart failure, cor pulmonale, arrhythmia) Willing to wear pap for the day after, noncomplex narcotics, no witnessed apneas or desaturations or airway obstruction	22.0% Desaturations	22% Desaturations	Bias with removal of patients admitted for non-OSA related reasons, no further complications than desaturation for those admitted, uses defined selection criteria and discharge criteria, did not find that BMI or AHI made a significant difference
Spiegel and Raval^28^	Selection of outpatient and inpatients are based on discharge, not intention, surgeon/anesthesiologist Surgeon/anesthesiologist	N/A	No outpatient complications 2.8% Desaturations 1.9% Postobstructive pulmonary edema	Bias due to the selection of outpatient and inpatients are based on discharge, not intention
Stefanescu et al^30^	Surgeon/anesthesiologist Surgeon/anesthesiologist	N/A	0.9% Hemorrhage	Only reports infectious and bleeding complications
Strocker et al^29^	Surgeon/anesthesiologist Surgeon/anesthesiologist	100.0% Obesity-related comorbidities	2.5% Obesity-related comorbidities	Small sample size, shows variety in respiratory admissions
Wu et al^31^	Surgeon/anesthesiologist Surgeon/anesthesiologist	100.0% Hypercapnic respiratory failure	1.3% Hypercapnic respiratory failure	No complications noted for those admitted overnight

Abbreviations: AHI, apnea-hypopnea index; BMI, body mass index; COPD, chronic obstructive pulmonary disease; ER, emergency room; N/A, not applicable; OSA, obstructive sleep apnea; PACU, postanesthesia care unit.

Eleven studies had some variant of NPP surgery involving 4261 patients with OSA.^21-31^ Five studies examined 1453 inpatients with OSA.^21,24-26,28^ Among the 9 studies that reported age, the average of outpatients with OSA was 42.5 years.^21-27,29,31^ This compared closely with an average age of 40.3 years for inpatient controls. Six studies reported outpatient BMI with an average BMI of 31.5^23-27,29^ and with inpatient controls averaging 32.0.^24-26^ Three studies reported apnea-hypopnea index (AHI) with an average value of 27.2.^24,27,29^ Two additional studies focused on respiratory disturbance index (RDI), reporting average RDI values of 35.0 and 36.0.^23,25^

NPP surgeries reported an average unplanned admission rate of 5.4% (1.3%-22.0%).^23,27,29,31^ One instance of intensive care unit (ICU) admission due to hypercapnic respiratory failure was noted, likely medication-related^31^ (Table 5). Three studies highlight a selection criterion that assessed patients based on cardiorespiratory comorbidities and utilized discharge protocols centered on respiratory and anesthetic considerations.^23,27,29^ In contrast, other studies relied on the discretion of anesthesiologist/surgeon. Rotenberg et al study reported an exceptionally high admission rate of 22%, which was markedly higher than other studies, with the next highest being 2.5%.^27^ This disparity can be attributed to Rotenberg et al’s stringent discharge criteria, which mandated 0 prolonged desaturation.

Eight NPP surgery studies reported a 24 hour complication rate of 9.2% among outpatients.^8,23-25,28-31^ Notably, one of these studies conducted a statistical analysis and did not find a statistically-significant difference between outpatient and inpatient complication rate^24^ (20.3% vs 30.3%, *P* = .09). Respiratory events stood out as common complications, including a particularly-severe instance of a hypercapnic respiratory failure.^31^ The rest were desaturation events, reported to occur in the outpatient population in 3 of the 8 studies.^23,24,27^ Two of these studies only reported respiratory complications requiring treatment or admission.^23,27^ Kandasamy et al do not find a significant difference between desaturation rates between outpatients and inpatients in the PACU (4.1% vs 6.0%, *P* = .5). However, a significant difference does emerge over a 24 hour window (5.2% vs 15.7%, *P* = .008). This is likely due to outpatients not being monitored overnight unlike inpatient counterparts.^24^ Studies presented varying viewpoints on factors influencing the risk of complications, with Kandasamy et al associating BMI (OR = 2.70, 95% CI = 1.48-4.91) and AHI (OR = 2.21, 95% CI = 1.166-4.188) with PACU desaturation risk, contrary to Rotenberg et al.^23,24,27^ Desaturation criteria varied across studies from below 85% to 94% (Table 5). Furthermore, bleeding, a concerning complication of uvulopalatopharyngoplasty (UPPP), was reported in only 1 outpatient.^30^

## Discussion

Concerns about ASC involve unexpected hospitalizations and potential for severe complications needing immediate hospital transfer. High rates of unplanned admissions following ASC surgery could discourage their use due to patient safety risk and the financial burden of many hospital transfers and thus will be a vital variable in determining whether a certain procedure will be able to be performed in an ASC context. To our knowledge, this review is the first published study to demonstrate whether standard NPP OSA surgery can be safely performed in an ASC setting.

Among all the studies reviewed, the medical cause admission rate was 5.4% among all intended outpatients with OSA. Notably, this stands in contrast to Tan et al, which found an admission rate of 50.2%. This discrepancy is likely due to Tan et al focusing on day-case discharges as opposed to surgeries explicitly intended for outpatients.^9^ In terms of severe complications in the early-postoperative period, there was 1 complication leading to an ICU transfer among 279 cases (0.3% rate).^31^ Kandasamy et al reported a higher 0.9% major respiratory complications rate for all patients, inpatient or outpatient.^24^ Inpatient studies, however, find similar results as this review. Rotenberg et al looked at 121 patients admitted overnight after sleep apnea surgery and found no severe complications.^8^ Mickelson and Hakim analyzed 347 patients and reported 4 patients transferred to the ICU (1.2%).^32^ Woodling et al looked at high-risk patients with OSA and OSA undergoing upper airway surgery and reported no patients that had severe complications overnight or in the PACU.^33^ Any severe complications that were reported within these studies occurred within 3 hours of the surgery. Thus, serious complications for upper airway surgery are not only infrequent but would occur during a timeframe that would allow for intervention within the ASC. Furthermore, there is a lower rate of serious complications within this review in comparison with inpatient studies.

On the surface, a 5.4% admission rate appears too elevated for an ASC setting, possibly making the costs too steep to sustain viable ASC operations. Yet, this percentage might be inflated. Rotenberg et al, which had the highest admission rate of 22%, mandated admission for any desaturation.^27^ However, the approach of admitting all desaturations may not be optimal as Kandasamy et al all reports desaturations within their outpatient population.^24^ Furthermore, many of these studies had their complications taken from their indications for admissions. This would indicate that many of these studies’ populations likely had desaturations but were not admitted and thus not reported within our review. Furthermore, Kandasamy et al and Rotenberg et al found no serious overnight complications for patients admitted due to minor desaturations in the PACU. This suggests that, despite PACU desaturations, severe complications are infrequent.^24,27^ Kandasamy et al also observed no meaningful distinction in PACU desaturation rates between outpatient and inpatient groups. This points to the idea that admission decisions in a hospital outpatient environment seem to be more influenced by patient factors.^24^ Furthermore, the studies in this review offer varied perspectives on the impact of BMI and AHI on desaturation risk.^23,24,27^ ASCs typically cater to patients with fewer comorbidities, lower BMI, and reduced AHI. Thus, if these procedures were performed in an ASC context rather than an outpatient hospital with healthier patients, it may have resulted in fewer desaturations.

The combination of these ideas indicates that patients with minor or intermittent desaturations could safely forgo oxygen supplementation, maintain stable vitals, and consequently be discharged. Moreover, it is important to keep in mind that patients with OSA have been desaturating during sleep for years, so these desaturation events following surgery might not indicate a significant abnormality for the individual. Patients should be monitored in PACU for hypoxemia, and it should be deemed fit for discharge as per standard anesthetic and recovery guidelines including an assessment for hypoxemia. If hypoxemia persists, a transfer to a higher care level facility may be warranted. Thus, Rotenberg et al’s approach of admitting patients based purely on desaturation probably skews the admission rates upward.^27^ As a result, our reported 5.4% is likely to be an overestimate. Although current studies do not pinpoint the exact proportion of patients who can comfortably cease oxygen supplementation and still sustain adequate oxygen saturation, the bulk of the data leans in this favorable direction. Taking into account all these elements, it is likely that NPP surgeries in this population might experience <3% admission rate due to medical reasons. This becomes clear when the study by Rotenberg et al is excluded; our rate of unplanned admissions falls significantly below 3%. This figure aligns with admission rates observed for other procedures.^15-17^

This review found a 3.8% incidence of respiratory complications, the vast majority being simple desaturations. All severe respiratory complications occurred within 4 hours post-surgery.^24,31^ However, the rate is unclear, as some studies classified patients based on discharge outcome, which infers a selection bias as outpatients are then less likely to suffer from complications. Furthermore, studies such as Hathaway and Johnson only reported admission-causing complications.^23^ This respiratory complication rates align with inpatient studies such as Rotenberg et al, which reported a similar 3.4% rate following OSA NPP surgeries.^8^ Other studies, such as Gessler and Bondy and Tan et al, reported a higher complication rate of 6.2% and 4.9%, respectively.^9,34^ Hemorrhage is another critical consideration, with only 1 reported hemorrhage within 24 hours in this review. (0.02%).^30^ This is a significantly-lower rate than in literature, such as Kim et al of 7.8%.^35^ The lower rate within this review could reflect advancements in surgical techniques, but potential errors from studies that include inpatient population must be considered, like Kandasamy et al’s reclassification of intended outpatients to inpatient in the case of a PACU hemorrhage.^24^ Kandasamy et al also indicate that if a hemorrhage does not occur within a PACU, any future hemorrhages will not be within the first postoperative night. The errors within these data make it difficult to make any conclusions on the rates of 24 hour complications of OSA surgery within an ASC. However, severe complications and hemorrhage occur at a low rate that would be feasible within an ASC setting. Current evidence suggests that outpatient OSA surgery does not increase the risk of 30 day complications compared with inpatient surgery.^21,26^

While upper airway surgery presents as a viable option for the ASC environment, the concern becomes the selection of patients. The current intended outpatient studies offer conflicting advice on mitigating immediate postoperative complications that lead to admissions. Rotenberg et al compared AHI and BMI between admitted and discharged patients and found no significant difference. However, the sample size was likely underpowered.^27^ Hathaway and Johnson reported that admitted and discharged patients had similar characteristics. However, both Hathaway and Johnson and Strocker et al conducted no statistical analysis to compare admitted and discharged patients.^23,29^ Outpatient-inpatient studies have mixed results as outpatients tend to have a lower BMI in both Rosero and Joshi and Kieff and Busaba.^25,26^ However, Kandasamy et al report that post-PACU complications are not affected by BMI, but those with a combination of AHI over 22 and BMI over 30 were more likely to desaturate in the PACU.^24^ Inpatient studies add more complexities as Kim et al report a higher immediate postoperative complication rate with higher AHI.^35^ Kezirian et al looked at 30 day complications and found a higher severe complication rate with AHI and BMI.^36^ However, there does seem to be a consensus that comorbidities lead to increased complications among the literature.^24,35,36^ The disagreement between different literature pieces creates an interesting dilemma on what factors to consider for admission. Number of comorbidities and control of comorbidities is an important factor to consider. Due to the lack of data of sleep surgery performed in the ASC environment, it would be advisable that caution be taken when selecting patients with higher BMI, AHI, and age. The last consideration is multilevel surgery. Inpatient studies have found that multilevel surgery not only increases 30 day complications but rates of serious complications within 30 days.^36,37^ However, this does necessarily translate to ASCs as these studies do not specifically analyze early-postoperative complications. In this review, Kandasamy et al did not find a statistically-significant difference in complications when UPPP includes additional procedures.^24^ Rotenberg et al used multilevel surgery patients and reported no serious complications.^27^ However, both of the aforementioned studies might not have a sufficiently-large enough sample size to find serious complications or a statistically-significant difference. As found in the previous factors, multilevel surgery seems to have conflicting evidence. As such, multilevel surgery should be performed on healthier patients in an ASC environment compared with stand-alone procedures performed to treat OSA.

With all considerations of admissions due to medical causes and severe complications, upper airway NPP surgery can be safely performed in OSA patients within an ASC context without sacrificing the cost-effectiveness of the ASC model. While the exact complication rate would be difficult to determine, these complications, such as desaturations, can be easily handled within an ASC context.

Our review has several limitations. First, the most common complication of OSA patients undergoing surgery was oxygen desaturation in PACU. However, these patients have been desaturating nightly for years preoperatively, using this as a complication measure is likely inflating the complication rate. Additionally, the lack of high-volume intended OSA outpatient studies, questions our findings applicability to the wider OSA population. Second, our review lacked a meta-analysis for a more quantitative assessment of the evidence. Without data aggregation across studies, our data are less robust and should be interpreted with due caution. A significant limitation of our review was the predominance of retrospective studies, which are prone to biases and errors such as selection bias. Moreover, complication reporting in many studies had unclear or incomplete data, leading to the use of surrogate measures like indications for unplanned admissions, introducing uncertainty. Additionally, it is unclear whether 30 day readmissions were a direct consequence of the surgery itself or unrelated causes and underreporting may exist as most studies only retrieve data from 1 hospital, overlooking patients who may have been readmitted elsewhere. The absence of detailed and consistent complications reporting limits the reliability of our findings. The heterogeneity across study methodologies and patient populations complicates the task of drawing firm conclusions and highlights the need for standardized approach in future research. Lastly, our review is limited by the studies that are currently available; unpublished studies or unindexed studies could alter our conclusions. Additionally, the absence of direct ASC studies, requiring us to use outpatient data as a proxy, impacts the validity of our study.

## Conclusion

When analyzing medical causes for admission and severe complications, NPP surgery can be feasibly and safely performed in an ASC environment as long as an oxygen discontinuation trial is performed on patients with desaturations or requiring oxygen supplementation. In general, the data support that healthier nonobese patients with moderate-to-severe OSA are favored over severe and/or obese patients with OSA for surgery at an ASC, but cases should be assessed on their individual merits for a risk/benefit analysis.

## Supplemental Material

sj-docx-1-ohn-10.1177_19160216251379325 – Supplemental material for Systematic Review on the Safety of Outpatient Upper Airway Surgery for Obstructive Sleep Apnea Patients in Ambulatory Surgical Centers Supplemental material, sj-docx-1-ohn-10.1177_19160216251379325 for Systematic Review on the Safety of Outpatient Upper Airway Surgery for Obstructive Sleep Apnea Patients in Ambulatory Surgical Centers by Muaaz Asghar, Kenny Pang, Mauz Asghar and Brian Rotenberg in Journal of Otolaryngology - Head & Neck Surgery
