# Supplementary material for: Systematic Review on the Safety of Outpatient Upper Airway Surgery for Obstructive Sleep Apnea Patients in Ambulatory Surgical Centers
Source: J Otolaryngol Head Neck Surg. 2025 Oct 29;54:19160216251379325. doi: 10.1177/19160216251379325 (PMC12576096; doi:10.1177/19160216251379325)
Supplement: sj-docx-1-ohn-10.1177_19160216251379325 – Supplemental material for Systematic Review on the Safety of Outpatient Upper Airway Surgery for Obstructive Sleep Apnea Patients in Ambulatory Surgical Centers [file sj-docx-1-ohn-10.1177_19160216251379325.docx]

**S1 Table :** Ovid-MEDLINE Search Results

| Query | Search Terms | Results |
| --- | --- | --- |
| #1 | Sleep Apnea Syndromes/ OR Sleep Apnea, Obstructive/ OR sleep apnea.mp. OR osa.mp. OR sleep apnoea.mp. OR osahs.mp. OR osas.mp. | 59,748 |
| #2 | Ambulatory Surgical Procedures/ OR Outpatients/ OR Surgicenters/ OR Ambulatory Care/ OR Ambulatory Care Facilities/ OR Ambulatory Care Sensitive Conditions/ OR ambulatory.mp. OR outpatient.mp. OR same day surgery.mp. OR same day discharge.mp. OR ambulatory surgical center.mp. OR ambulatory surgery center.mp. OR hopd.mp. OR day case surgery | 337,671 |
| #3 | #1 AND #2 | 2495 |

**S2 Table:** Ovid-Embase Search Results

| Query | Search Terms | Results |
| --- | --- | --- |
| #1 | sleep disordered breathing/ OR upper airway resistance syndrome/ OR sleep apne*.mp.OR sleep apnoe*.mp OR (obstruct* adj2 hypopnea*).mp. OR (obstruct* adj2 hypopnoea*).mp. OR (obstruct* adj2 hypoapnea*).mp OR (obstruct* adj2 hypoapnoea*).mp OR (sleep disorder* adj1 breathing).mp OR osa.mp OR osas.mp. OR osahs.mp. | 115,385 |
| #2 | ambulatory surgery/ OR outpatient care/ OR outpatient/ OR outpatient department/ OR outpatient.mp. OR (same day adj2 surg*).mp. OR ambulatory.mp. OR day surgery.mp. OR (day-case adj2 surg*).mp. OR (same-day adj2 discharg*).mp. | 595,339 |
| #3 | #1 AND #2 | 5435 |
| #4 | limit #3 to "remove medline records" | 2924 |

**S3 Table:** Scopus Search Results

| Query | Search Terms | Results |
| --- | --- | --- |
| #1 | ( TITLE-ABS-KEY ( "sleep apne*" ) OR TITLE-ABS-KEY ( "osa" ) OR TITLE-ABS-KEY ( "osas" ) OR TITLE-ABS-KEY ( "osahs" ) OR TITLE-ABS-KEY ( "obstruct*" W/2 "hypopnea*" ) OR TITLE-ABS-KEY ( "obstruct*" W/2 "hypopnoea*" ) OR TITLE-ABS-KEY ( "obstruct*" W/2 "hypoapnea*" ) OR TITLE-ABS-KEY ( "obstruct*" W/2 "hypoapnoea*" ) OR TITLE-ABS-KEY ( "obstruct*" W/2 "hypo-apnea*" ) OR TITLE-ABS-KEY ( "obstruct*" W/2 "hypo-apnoea*" ) OR TITLE-ABS-KEY ( "sleep disorder*" W/1 "breathing" ) OR TITLE-ABS-KEY ( "sleep apno*" ) ) AND (( TITLE-ABS-KEY ( "ambulatory" ) OR TITLE-ABS-KEY ( "outpatient" ) OR TITLE-ABS-KEY ( "day surgery" ) OR TITLE-ABS-KEY ( "day-case" ) OR TITLE-ABS-KEY ( "same day" W/2 "surg*" ) OR TITLE-ABS-KEY ( "same-day" W/2 "discharg*" ) OR TITLE-ABS-KEY ( "day case" ) OR TITLE-ABS-KEY ( "daycase" ) OR TITLE-ABS-KEY ( "day patient" ) ) .  ( ( TITLE-ABS-KEY ( "sleep apne*" ) OR TITLE-ABS-KEY ( "osa" ) OR TITLE-ABS-KEY ( "osas" ) OR TITLE-ABS-KEY ( "osahs" ) OR TITLE-ABS-KEY ( "obstruct*" W/2 "hypopnea*" ) OR TITLE-ABS-KEY ( "obstruct*" W/2 "hypopnoea*" ) OR TITLE-ABS-KEY ( "obstruct*" W/2 "hypoapnea*" ) OR TITLE-ABS-KEY ( "obstruct*" W/2 "hypoapnoea*" ) OR TITLE-ABS-KEY ( "obstruct*" W/2 "hypo-apnea*" ) OR TITLE-ABS-KEY ( "obstruct*" W/2 "hypo-apnoea*" ) OR TITLE-ABS-KEY ( "sleep disorder*" W/1 "breathing" ) OR TITLE-ABS-KEY ( "sleep apno*" ) ) AND ( TITLE-ABS-KEY ( "ambulatory" ) OR TITLE-ABS-KEY ( "outpatient" ) OR TITLE-ABS-KEY ( "day surgery" ) OR TITLE-ABS-KEY ( "day-case" ) OR TITLE-ABS-KEY ( "same day" W/2 "surg*" ) OR TITLE-ABS-KEY ( "same-day" W/2 "discharg*" ) OR TITLE-ABS-KEY ( "day case" ) OR TITLE-ABS-KEY ( "daycase" ) OR TITLE-ABS-KEY ( "day patient" ) ) ) | 3721 |

**S4 Table**: Cochrane Search Results

| Query | Search Terms | Results |
| --- | --- | --- |
| #1 | MeSH descriptor: [Sleep Apnea Syndromes] explode all trees | 3793 |
| #2 | "sleep apnea" OR "sleep apnea hypopnea syndrome" OR "sleep apnea-hypopnea syndrome" or "sleep apnea syndrome" OR "sleep-disordered breathing" OR "sleep-disordered breathing" OR "sleep apnoea*" OR "sleep apnoea hypopnoea syndrome" OR "sleep apnoea syndrome" OR "sleep apnoea-hypopnoea syndrome" OR "OSA" OR "OSAS" OR "OSAHS" | 9433 |
| #3 | obstruct* NEAR/2 "hypopnea" | 95 |
| #4 | obstruct* NEAR/2 hypopnoea | 92 |
| #5 | MeSH descriptor: [Ambulatory Surgical Procedures] explode all trees | 1717 |
| #6 | MeSH descriptor: [Outpatients] this term only | 1877 |
| #7 | MeSH descriptor: [Ambulatory Care Facilities] this term only | 764 |
| #8 | MeSH descriptor: [Ambulatory Care] this term only | 3562 |
| #9 | MeSH descriptor: [Surgicenters] this term only | 8 |
| #10 | "ambulatory" OR "outpatient" OR "same day surgery" OR "same-day surgery" OR "daycase" OR "day-case" OR "day-case" | 64,831 |
| #11 | "same day" NEAR/2 discharg* OR "day-case" NEAR/2 surg* OR "same day" NEAR/2 surg* | 908 |
| #12 | #1 OR #2 OR #3 OR #4 | 9478 |
| #13 | #5 OR #6 OR #7 OR #8 OR #9 OR #10 OR #11 | 65,636 |
| #14 | #12 AND #13 | 667 |
| #15 | Selected only CENTRAL trials from #14 | 591 |
